# Arbuscular Mycrorrhizal Fungi Inoculation and Applied Water Amounts Modulate the Response of Young Grapevines to Mild Water Stress in a Hyper-Arid Season

**DOI:** 10.3389/fpls.2020.622209

**Published:** 2021-01-14

**Authors:** Nazareth Torres, Runze Yu, Sahap Kaan Kurtural

**Affiliations:** Department of Viticulture and Enology, University of California, Davis, Davis, CA, United States

**Keywords:** climate change, water scarcity, grapevine physiology, berry metabolism, arbuscular mycorrhizal fungi, sustainable viticulture

## Abstract

Several factors may affect the success of a replanting vineyard. Given the current environmental conditions, an optimized irrigation schedule would still be one of the most desirable tools to improve crop productivity and fruit quality. On the other hand, the symbiosis of grapevines with arbuscular mycorrhizal fungi (AMF) is a key component of the vineyard production systems improving the vine growth, nutrient uptake, and berry quality. The aim of this study was to characterize the response of Merlot grapevines to AMF inoculation and two different irrigation amounts in their first productive year. The experiment was conducted on 2-year Merlot grapevines inoculated with AMF (I) or not-inoculated (NI) and subjected to two irrigation amounts, full irrigated (FI), where the amount of water was enough to maintain expansive growth and half irrigated (HI) where plants received the half of the amount of water of FI plants. Water status, gas exchange parameters, growth, mineral content, berry composition, and mycorrhizal colonization were monitored through the season. AMF inoculation improved the grapevine vegetative growth, water status, and photosynthetic activity, especially when vines were subjected to HI irrigation; however, no effect was observed on the leaf mineral content, must pH, total soluble solids, or total acidity. The main effects were observed on the flavonoid composition of berry skins at harvest. Irrigation amounts and mycorrhizal inoculation modified cyanidin and peonidin derivatives whereas flavonol composition was mainly affected by irrigation treatments. A strong relationship between the mycorrhizal colonization rate of roots and total quercetins, cyanidins, and peonidins was found. Findings support the use of a mycorrhizal inoculum and a better water management in a hyper-arid growing season; however, these results may be affected by edaphoclimatic characteristics and living microbiota in vineyard soils, which should be taken into account before making the decision of inoculating the vineyard.

## Introduction

Grapevine is an economically important crop worldwide with a global surface area of 7.45 million ha, which is mainly cultivated for wine making. California stands out as the fourth leading wine producer in the world with 257,784 ha of wine grapes and 4.28 million tons of grapes harvested in 2018, leading to an annual economic impact of $57.6 billion (Wine Institute, 2020). Nevertheless, winegrowers face the challenge of replanting their vineyards when grapevines are not producing due to diseases such as grapevine red blotch virus, trunk diseases, or other viral diseases such as leaf roll disease, or because the plant material is producing substandard fruit and consequently compromising the wine quality. However, several factors need to be taken into account when replanting, as improper establishment during this stage causes considerable economic loss to the industry.

Arbuscular mycorrhizal fungi (AMF) are soil-borne fungi that form mutualistic relationships with 80% of the superior plants (Smith and Read, 2008). In viticultural regions, the AMF-grapevine symbiosis was pointed out as a key component of the vineyard system (Trouvelot et al., 2015). Recent research suggested the key role that this symbiosis might play in facing environmental constrains (Torres et al., 2018b). The application of mycorrhizal inocula has emerged as a reliable technique to enhance the agricultural productivity whereas reducing environmental costs (Berruti et al., 2016; Hamilton et al., 2016). Frequently, these commercial inoculants consist of a single or few AM fungal isolates grown in plant culture or greenhouse conditions with annual grasses or forbs (Gianinazzi and Vosátka, 2004), hence they might not establish on woody grapevines that have different ecosystem preferences (Holland et al., 2018). It is well established that under controlled conditions AMF inoculation of grapevines promotes increased growth (Linderman and Davis, 2001), drought tolerance (Nikolaou et al., 2003), and nutrient uptake (Karagiannidis et al., 2007). Moreover, AMF protect grapevines grown in controlled conditions against pathogens through stimulation of key genes of the phenylpropanoid biosynthesis in leaves (Bruisson et al., 2016) and inhibit their transmission by impairing the growth of nematode vectors in roots and their reproduction in soils (Hao et al., 2018). Although it is widely accepted that AMF-grapevine association improves grapevine growth and mineral uptake in vineyards (Trouvelot et al., 2015), contradictory results were recently reported when studying the protective role of the symbiosis against pathogens such as *Ilyonectria* (Holland et al., 2019). Similarly, AMF inoculation may affect berry primary and secondary metabolism in response to environmental stresses when grapevines were cultivated under controlled conditions (Torres et al., 2016, 2018c) but little is known about their effect under natural conditions. Additionally, rootstock genotype and type of inoculum could also influence the effectiveness of mycorrhizal inoculation and therefore the response of young vines to the environment (Holland et al., 2018).

On the other hand, most wine grape producing regions are subjected to seasonal drought, but based on the global climate models an increase in aridity is predicted in the future. Hence, an optimized irrigation schedule would still be one of the most desirable tools to improve crop productivity and quality in historically non-irrigated viticulture areas where irrigation is expanded fast to mitigate environmental stress (Costa et al., 2016; Resco et al., 2016). In addition, in warm and hot viticultural regions such as California that rely on irrigation for crop production, water resources, especially groundwater, are becoming scarce due to extended drought periods and overuse by irrigated agriculture (Wilson et al., 2020).

Currently, winegrowers are aware of the importance of a sustainable viticulture that ensures the profitability in the future, without compromising berry quality. However, to the best of our knowledge little is known about the contribution AMF inoculation may have for implementing the effects of different irrigation amounts on the performance and berry quality of young grapevines under field conditions. Therefore, the aim of this study was to characterize the response of young Merlot grapevines to AMF inoculation subjected to two different irrigation amounts in their first productive year.

## Materials and Methods

### Plant Material and Experimental Design

This study was conducted in the Oakville Experimental Station (38.429°–122.410°). The vineyard was planted to Merlot clone 181 on 3,309 C rootstock in 2018 at 3 m × 2 m (row × vine) spacing in E–W orientation. The grapevines were spur pruned and trained to quadrilateral trellis system 1.38 m above vineyard floor with catch wires at 1.68 m. The experimental vineyard was drip-irrigated with one or two emitters spaced every 2 m along the drip line and with the capacity of deliver 3.8 L of water per hour. Natural vegetation was allowed to grow in alleys and mowed according to vineyard manager’s discretion, with a no-till system in place.

The experiment consisted in a 2 × 2 factorial design (AMF inoculated or not-inoculated vines subjected to two irrigation amounts) with four replications of seven grapevine plots arranged in a split plot design. The commercial Myco Apply Endo Maxx inoculum (Mycorrhizal Applications LLC, OR, United States) consisted in a suspendable powder containing living propagules of *Rhizophagus intraradices* (basionym *Glomus intraradices*), *Funneliformis mosseae* (basionym *Glomus mosseae*), *Glomus aggregatum*, and *Glomus etunicatum* containing 5,625 propagules/g. The mycorrhizal inoculum was diluted in water to final concentration of 5.3 mg/L in order to achieve the manufacturer’s recommended rate of 10 g each 1,000 plants. The diluted AMF inoculum was applied in-field drench during 50 s around the trunk of each vine at the beginning of the growing season (20 March) by using a 56 L spot sprayer. Although the inoculum manufacturer did not report other microorganisms accompanying AMF^1^, it is known that commercial AMF inocula, obtained following industrial production processes, are home of a large and diverse community of bacteria with important functional plant promoting growth traits, that may act in synergy with AMF providing additional services and benefits (Agnolucci et al., 2019). Therefore, non-inoculated vines received the same amount of a filtrate inoculum with the objective of restoring rhizobacteria and other soil free-living microorganism accompanying AMF and that play an important role in the uptake of soil resources as well as in the infectivity and efficiency of AMF isolates (Agnolucci et al., 2015). The filtrate was obtained by passing diluted mycorrhizal inoculum through a Whatman filter paper Grade 5 with particle retention of 2.5 μm (Whatman 5; GE Healthcare, MA, United States). Phosphorus amounts in the vineyard soil was measured before the experiment and was low, thus, that phosphorus level (<10 mg/kg) was sufficient to ensure adequate development of non-inoculated plants, even under water deficit and not excessive enough to decrease the mycorrhizal diversity in the vineyard and thereby the root colonization (Van Geel et al., 2017). Irrigation treatments started at the beginning of summer (May 2020) until harvest (August 2020). Vineyard crop evapotranspiration (ET_c_) was calculated by multiplying the reference evapotranspiration (ET_o_, CIMIS #77) and the crop coefficient (*K*_c_). Thus, half of the inoculated and non-inoculated vines were irrigated to ensure the full of expansive growth that corresponded with the amount of water needed to restore the 100% of the ET_c_ (Full irrigated, FI). The other half of inoculated and non-inoculated vines received half of the amount of water received by FI plants (half irrigated, HI). Irrigation was applied weekly. Each treatment had four replicates consisting in 7 grapevines, 3 of which were sampled and the 4 on distal ends were treated as border plants.

### Weather Conditions

**FIGURE 1:**
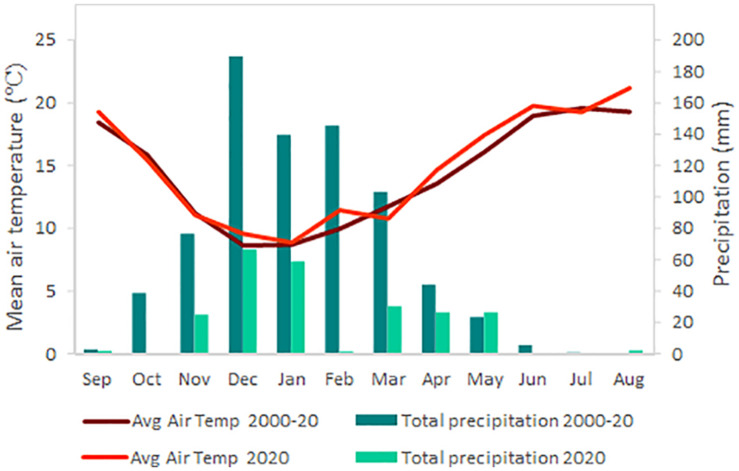
Average air temperature (Avg air temp) and precipitation during the growing season 2019–2020 and the average for the same period in the last 20 years (2000–2020). Weather data were obtained from the CIMIS weather station #77 (Oakville, CA, United States) located at the research site.

Weather data (Figure 1) were obtained from the California Irrigation Management Information Systems, CIMIS, station (#77, Oakville, California) located on site during the growing season covered by the trial and the reference period 2000 to 2020 (California Department of Water Resources, 2020).

### Plant Water Status and Leaf Gas Exchange Parameters

Plant water status was measured as stem water potential (SWP) every 2 weeks during the growing season around the solar noon. A fully-expanded leaf per treatment-replicate exposed to sun and without signs of disease and/or damage was selected and covered 2 h before measurements with a foil-lined zip-top plastic bag in order to suppress transpiration. Then, the SWP was directly determined with a pressure chamber (Model 610 Pressure Chamber Instrument, PMS Instrument Co., Corvallis, OR, United States).

Coinciding with the main phenological events, leaf gas exchange was measured beginning at solar noon on one fully expanded leaf with a CIRAS-3 portable photosynthesis system (PP Systems, Amesbury, MA, United States) equipped with a leaf chamber with a 4.5 cm^2^ window. The window of the chamber was oriented perpendicularly toward the sun to allow for saturation light conditions (1984 ± 52 μmol/m/s). Reference CO_2_ was set to 390 μmol/mol CO_2_ at a flow rate of 200 mL/min. Leaf gas exchange was performed, leaving the cuvette for 40–60 s until reaching a steady state and measurements were taken in triplicate.

### AMF Colonization and Relative Mycorrhizal Dependency Index

Intraradical AMF colonization was estimated before treatment application (native colonization, 20 March), 3 months after treatment application (25 June), and at harvest (26 August). Root samples (mainly hair roots) from three grapevines per replicate were collected at a depth of 15 and 20 cm away from the vine trunk by using a fork, and stored in zip bags for further analysis. Then, each replicate root sample was washed with water in the sink, cleared, and stained according to methods described in Koske and Gemma (1989). AMF colonization was determined by examining 1-cm root segments (50 per treatment/replicate) under the microscope (Supplementary Figure 1). Then, intensity of the intraradical mycorrhizal colonization was calculated for each treatment/replicate as described previously by Torres et al. (2016). Briefly, the extension of mycorrhizal colonization was determined by estimating the product of the mycorrhizal colonization in width and length according to a scale range between 0 and 10 where 0 is complete absence of fungal structures. The extension of each treatment/replicate was calculated as the sum of the product of mycorrhizal colonization in width and length divided to the number of root segments. Then, the incidence of mycorrhizal colonization was estimated by dividing the number of root segments with presence of fungal structures and the total observed segments. Finally, the intensity of the colonization was calculated as the product between the extension and incidence, and the result was expressed as percentage of colonization.

Relative mycorrhizal dependency (RMD) index was calculated following Bagyaraj (1994): RMD = Leaf fresh weight of I vines × 100/Leaf fresh weight of NI vines. This index allows establishment of the crop dependency upon the mycorrhizal symbiosis for reaching its maximum growth for given environmental conditions.

### Mineral Composition of Leaf Blades

During the growing season (05 June) two leaves per vine/replicate were collected, petioles were removed, and leaf blades were dried at 70°C in an oven. Then, mineral analysis was carried out by using couple plasma-mass spectrometry by Dellavalle, Inc. (Fresno, CA, United States). Nitrogen (N) was determined via automated combustion analysis (method B-2.20) while phosphorus (P), potassium (K), sodium (Na), calcium (Ca), magnesium (Mg), zinc (Zn), manganese (Mn), boron (B), iron (Fe), and cuprum (Cu) were analyzed via Nitric/Perchloric Acid Digestion (method B-4.20) as described by Gavlak et al. (1994).

### Canopy Architecture, Grapevine Growth, and Yield Components

All the growth parameters were measured on the three middle vines in each replicate and the values were averaged for the replicate value. Green pruning was carried out before the cluster development (06 May) to avoid the excessive vegetative growth and ensure a good balance between the growth of vegetative and reproductive organs of the grapevines. Removed shoots from the three middle grapevines were weighed. Trunk diameter was measured with a carbon fiber composite digital caliper (Fisher Scientific, Waltham, MA, United States). At harvest, leaves were removed and leaf area was measured with a LI-3100 Area meter (LI-COR, Lincoln, NE, United States). Clusters were harvested and weighted to obtain the yield per vine. Measurements were performed on the three middle grapevines within each replicate and averaged.

### Berry Size and Composition

Thirty berries were randomly collected from the middle vines within each replicate and immediately processed. Berries were weighed and gently pressed by hand to squeeze the juice. Total soluble solids (TSS) were determined using a temperature-compensating digital refractometer (Atago PR-32, Bellevue, WA, United States). Must pH and titratable acidity (TA) were determined with an autotritrator (Metrohm 862 Compact Titrosampler, Herisau, Switzerland). TA was estimated by titration with 0.1 N sodium hydroxide to an end point of 8.3 pH and reported as g/L of tartaric acid.

### Berry Skin Flavonoid Composition

For flavonoid analysis 20 berries were randomly collected from each treatment-replicate and after gently peeling, skins were freeze-dried (Cold Trap 7385020, Labconco, Kansas City, MO, United States). Dried tissues were ground with a tissue lyser (MM400, Retsch, Germany). Fifty mg of the resultant powder was extracted in methanol: water: 7 M hydrochloric acid (70:29:1, V:V:V) to simultaneously determine flavonol and anthocyanin concentration and profile as previously described by Martínez-Lüscher et al. (2019). Briefly, extracts were filtered (0.45 μm, Thermo Fisher Scientific, San Jose, CA, United States) and analyzed using an Agilent 1260 series reversed-phase high performance liquid chromatography (HPLC) system (Agilent 1260, Santa Clara, CA, United States) coupled to a diode array detector. Separation was performed on a reversed-phase C18 column LiChrospher^®^ 100, 250 mm × 4 mm with a 5 μm particle size and a 4 mm guard column of the same material at 25°C with elution at 0.5 mL per minute. The mobile phase was designed to avoid co-elution of anthocyanins and flavonols (Martínez-Lüscher et al., 2019) and consisted in a constant 5% of acetic acid and the following gradient (v/v) of acetonitrile in water: 0 min 8%, at 25 min 12.2%, at 35 min 16.9%, at 70 min 35.7%, 65% between 70–75 min, and 8% between 80–90 min. The identification of flavonoid compounds was conducted by determining the peak area of the absorbance at 280, 365, and 520 nm for flavan-3-ols, flavonols, and anthocyanins, respectively. Identification of individual flavan-3-ols, anthocyanins, and flavonols were made by comparison of the commercial standard retention times found in the literature. Commercial standards of epicatechin, malvidin-3-*O*-glucoside, and quercetin-3-*O*-glucoside (Sigma-Aldrich, St. Louis, MO, United States) were used for the quantification of flavan-3-ols, anthocyanins, and flavonols, respectively.

### Labor Operation Costs, Gross Income, and Water Footprint of Irrigation Systems and AMF Inoculation

Cost estimates on labor operations and gross income per hectare were calculated based on yield and net returns per hectare (Kurtural et al., 2020). Water footprint (WF) was calculated as described by Zotou and Tsihrintzis (2017). Briefly, for the green component of the WF (green WF), precipitation data during the growing season was obtained from the CIMIS Station (#77, Oakville, CA, United States) and estimated as m^3^/ha to obtain the total green consumed water volume (green CWU). Then the value was divided by the yield expressed as ton/ha. The blue component of the WF (blue WF) was calculated with the total irrigation water amount that grapevines received per hectare, and this blue consumed water volume (blue CWU) value was divided by the yield (ton/ha). The gray component of the WF was not calculated given that our experimental conditions avoided the use of fertilizers. Then, the total WF was estimated as the sum of green WF and blue WF.

### Statistical Analysis

Statistical analyses were performed in R-Studio version 3.6.1 (RStudio: Integrated Development for R., Boston, MA, United States) for Windows. All the monitored parameters were fit in linear mixed-effect models (LMEM) by using the lmer function from lme4 package (Bates et al., 2020) with AMF inoculation (M), irrigation treatment (I), and their combination (M × I) as fixed factors, and replicate as random factor (Bates et al., 2015). The significance of the models was tested with the lmerTest package (Kuznetsova et al., 2020). Then, pairwise contrasts were conducted with function lsmeans from lsmeans package (Lenth, 2018) using the Kenward–Roger method and Tukey adjustment for *p*-values. Previously, for gas exchange parameters, stem water potential, mycorrhizal colonization, and flavonoid contents a mixed-effect model including sampling date (T) as fixed factor was run (Supplementary Tables 1–3). However, as the treatment effect seemed to be independent in the sampling date (with the exception of WUE and quercetin-3-glucoside content), sampling date was removed from the analysis to assess the effect of treatments for each sampling date. Finally, correlations between the percentage of mycorrhizal inoculation and flavonoid contents were calculated with the Pearson’s test using the same software.

## Results

### Weather Conditions, Mycorrhizal Colonization, and Grapevine Performance

The comparison between the growing season of the experiment and the reference data for the same period within the last 20 years showed that 2019–2020 was warmer and drier (Figure 1). Thus, average daily temperature was 0.5°C higher, especially in August, which reached 1.8°C more, and precipitation of 530 mm less compared to the average, hence, the 2020 growing season was an extreme year regarding temperature and rainfall.

Native mycorrhizal colonization was determined before treatment application and no differences between them were observed (Table 1). The mycorrhizal colonization intensity was analyzed (Supplementary Figure 1) after 3 months of treatment application to ensure the establishment of the mycorrhizal symbiosis, which frequently take place after 2–4 months of inoculation. Similar patterns in AMF colonization intensity were observed in both, 3 months after inoculation and at harvest, where roots from inoculated grapevines showed percentages of colonization values threefold higher than non-inoculated ones (Table 1). In addition, we observed increased AMF colonization rates along the growing season as shows the significant effect of the sampling date (T, *p* ≤ 0.0001, Supplementary Table 1) and its interaction with the AMF inoculation (M × T, *p* ≤ 0.0001, Supplementary Table 1).

**TABLE 1:** Mycorrhizal colonization at the beginning of the season, 3 months post-treatment application, and at harvest and relative mycorrhizal dependency (RMD) for vegetative growth of Merlot/3309C grapevines subjected to different irrigation amount (FI, Full Irrigated; HI, Half Irrigated), AMF inoculation (I, inoculated; NI, non-inoculated), and their combinations.

	Mycorrhizal colonization (%)			RMD (%)
	Native	After 3 months	Harvest	
**Treatments**				
FINI	1.68 ± 1.04	4.19 ± 1.16 b	14.14 ± 4 ab	78.43 ± 10.64 b
FII	1.11 ± 0.45	21.16 ± 2.66 a	26.28 ± 3.47 a	
HINI	2.21 ± 0.67	7.91 ± 0.76 b	9.23 ± 1.99 b	116.52 ± 8.54 a
HII	2.78 ± 0.67	16.38 ± 5.17 a	24.09 ± 4.02 a	
***LMEM***				
Irrigation (I)	*	ns	ns	*
Mycorrhizal inoculation (M)	ns	**	**	
I × M	ns	ns	ns	

Grapevines were measured in Oakville (California) during the 2020 growing season. Values represent means ± SE (n = 4) separated by Kenward–Roger method and Tukey’s p-value adjustment (P ≤ 0.05). Different letters within column, indicate significant differences as affected by Irrigation amount, I, AMF inoculation, M, and their interaction (I × M). ns, * and ** indicate non-significance or significance at 5%, and 1% probability levels, respectively. LMEM, linear mixed-effect model.

Relative mycorrhizal dependency (RMD) index allows assessing the dependency of a crop on the mycorrhizal symbiosis to achieve its maximum growth at a given environmental condition. Under FI conditions, RMD values were lower than 100% indicating that the mycorrhizal association impairs the vegetative growth of grapevines; however, RMD values for HI conditions highlighted the role of the mycorrhizal symbiosis for improving grapevine growth under water deficit conditions (Table 1).

Grapevine vegetative growth was also monitored during the 2020 growing season by measuring the green pruning weight, trunk diameter, and leaf area (Table 2). Measurements before treatment showed no differences between the different plants concerning trunk diameter (data not shown), corroborating the effect of treatments modulating vegetative growth of vines. Irrigation amount was the main factor affecting both vegetative growth and yield, with grapevines subjected to HI decreasing them (Table 2). However, as RMD reported AMF inoculation impair the grapevine growth estimated as trunk diameter and as green pruning weight when vines were FI, whereas under HI conditions, inoculated vines improved their growth (I × M, *p* ≤ 0.05). Finally, the leaf area to fruit ratio was not affected by treatments applied. The contents of minerals measured in leaf blades were not affected by AMF inoculation or applied water amount in our experiment (Table 3).

**TABLE 2:** Vegetative growth, yield, and leaf area to fruit ratio of Merlot/3309C grapevines subjected to different irrigation amounts (FI, Full Irrigated; HI, Half Irrigated), AMF inoculation (I, inoculated; NI, non-inoculated), and their combinations during the 2020 growing season (first productive year) in Oakville (California).

	Green pruning (kg/plant)	Trunk diameter (cm)	Leaf area (cm^2^)	Yield (kg/plant)	Leaf area to fruit ratio (m^2^/kg)
**Treatments**					
FINI	0.239 ± 0.023 a	1.44 ± 0.04 a	10498.0 ± 2980.3	0.317 ± 0.03	3.32 ± 0.91
FII	0.212 ± 0.016 ab	1.34 ± 0.04 ab	7402.8 ± 835.8	0.257 ± 0.03	3.00 ± 0.50
HINI	0.156 ± 0.015 b	1.19 ± 0.05 b	4694.4 ± 921.0	0.246 ± 0.01	1.89 ± 0.30
HII	0.185 ± 0.013 ab	1.30 ± 0.03 ab	5274.8 ± 857.9	0.209 ± 0.02	2.62 ± 0.56
***LMEM***					
Irrigation amount (I)	***	**	*	*	ns
AMF inoculation (M)	*	ns	ns	ns	ns
I × M	*	*	ns	ns	ns

Values represent means ± SE (n = 4) separated by Kenward–Roger method and Tukey’s p-value adjustment (P ≤ 0.05). Different letters within column, indicate significant differences as affected by ‘Irrigation amount, I,’ ‘AMF inoculation, M’ and their interaction (I × M). ns, *, **, and *** indicate non-significance or significance at 5, 1, and 0.1% probability levels, respectively. LMEM, linear mixed-effect model.

**TABLE 3:** Leaf mineral content of Merlot/3309C grapevines subjected to different irrigation amounts (FI, Full Irrigated; HI, Half Irrigated), AMF inoculation (I, inoculated; NI, non-inoculated) and their combinations during the 2020 growing season (first productive year) in Oakville (California).

	N	P	K	Zn	Mn	Na	Ca	B	Mg	Fe	Cu
	**%**	**%**	**%**	**mg/kg**	**mg/kg**	**%**	**mg/kg**	**%**	**%**	**mg/kg**	**mg/kg**

**Treatments**											
FINI	3.26 ± 0.09	0.26 ± 0.01	1.23 ± 0.12	472.0 ± 73.0	156.0 ± 24.0	0.018 ± 0.003	1.73 ± 0.18	77.50 ± 9.60	0.39 ± 0.02	220.25 ± 12.80	100.3 ± 34.7
FII	3.13 ± 0.06	0.26 ± 0.02	1.34 ± 0.03	389.8 ± 49.5	119.5 ± 12.4	0.010 ± 0.004	1.39 ± 0.08	62.25 ± 2.56	0.35 ± 0.02	201.75 ± 13.97	96.5 ± 41.8
HINI	3.26 ± 0.06	0.27 ± 0.01	1.28 ± 0.05	406.0 ± 52.5	134.5 ± 18.6	0.013 ± 0.003	1.51 ± 0.09	63.25 ± 9.32	0.38 ± 0.01	225.75 ± 10.25	122.0 ± 22.3
HII	3.25 ± 0.05	0.26 ± 0.02	1.29 ± 0.11	351.8 ± 32.9	123.8 ± 21.0	0.010 ± 0.004	1.48 ± 0.23	58.50 ± 6.46	0.37 ± 0.05	227.50 ± 31.67	122.0 ± 55.5
***LMEM***											
Irrigation amount (I)	ns	ns	ns	ns	ns	ns	ns	ns	ns	ns	ns
AMF inoculation (M)	ns	ns	ns	ns	ns	ns	ns	ns	ns	ns	ns
I × M	ns	ns	ns	ns	ns	ns	ns	ns	ns	ns	ns

*Values represent means ± SE (n = 4) separated by Kenward–Roger method and Tukey’s p-value adjustment (P ≤ 0.05). ns indicate non-significance. All values are expressed as % or mg of the mineral per kg of leaf dry weight. LMEM, linear mixed-effect model.*

### Plant Water Status and Gas Exchange Parameters During the Growing Season

**FIGURE 2:**
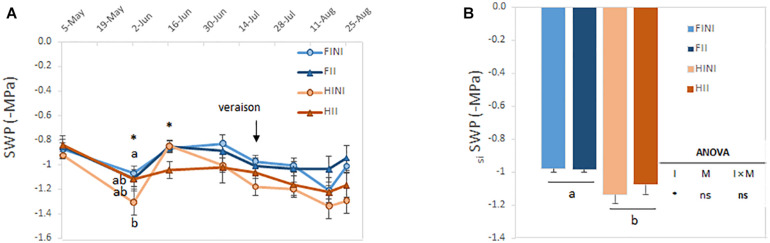
Mid-day stem water potentials (SWP) monitored every 2 weeks during the growing season **(A)** and seasonal integrals of the SWP (_si_SWP, **B**) of field grown Merlot/C3309 grapevines subjected to different irrigation amounts (FI, Full Irrigated; HI, Half Irrigated), AMF inoculation (I, inoculated; NI, non-inoculated), and their combinations. Values represent means ± SE (*n* = 4) separated by Kenward–Roger method and Tukey’s *p*-value adjustment (*P* ≤ 0.05). At each time point, different letters indicate significant differences as affected by Irrigation amount, I, AMF inoculation, M and their interaction (I × M) according to the linear mixed-effect model. ns and * indicate non-significance and significance at 5% probability levels, respectively.

Plant water status was determined by monitoring the SWP each 2 weeks at noon during the growing season. The SWP values ranged between −0.8 and −1.3 MPa at harvest (Figure 2A) suggesting that the amount of applied water was successful in reaching the SWP target during the growing season. Irrigation amount was the main factor affecting the water status of vines especially at the end of the growing season. However, before *veraison* AMF inoculation could increase the grapevine water status under HI conditions (I × M, *p* ≤ 0.05, Figure 2A). The calculation of the seasonal integral of SWP showed the same pattern; hence, _si_SWP was mainly affected by irrigation system with HI plants being the most stressed vines (Figure 2B).

**FIGURE 3:**
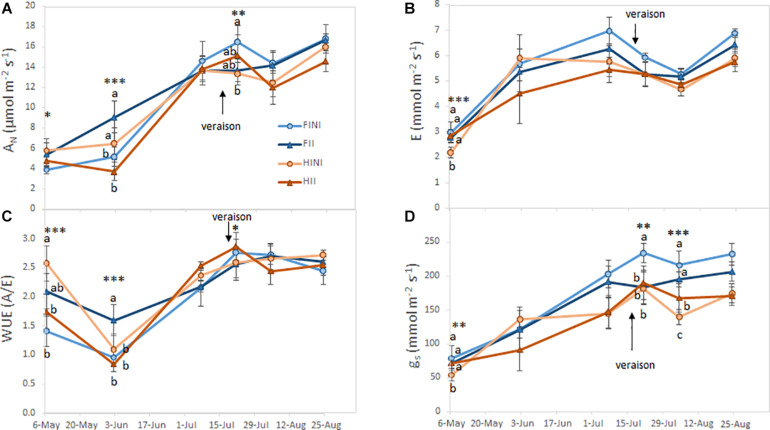
Net carbon assimilation (*A*_N_; **A**), leaf evapotranspiration (E; **B**), instantaneous water use efficiency (WUE; **C**), and stomatal conductance (*g*_s_; **D**) measured during the growing season of field grown Merlot/C3309 grapevine subjected to different irrigation amounts (FI, Full Irrigated; HI, Half Irrigated), AMF inoculation (I, inoculated; NI, non-inoculated) and their combinations. Values represent means ± SE (*n* = 4) separated by Kenward–Roger method and Tukey’s *p*-value adjustment (*P* ≤ 0.05). At each time point, different letters indicate significant differences as affected by Irrigation amount, I, AMF inoculation, M, and their interaction (I × M) according to the linear mixed-effect model. *, **, and *** indicate significance at 5, 1, and 0.1% probability levels, respectively.

Gas exchange parameters monitored during the season are shown in Figure 3. Carbon assimilation (*A*_N_) rates increased through the growing season, and were affected by the interaction between AMF inoculation and irrigation amounts (Figure 3A). Thus, FII plants showed the highest values of *A*_N_ at fruit set and harvest, while FINI grapevines increased A_N_ after *veraison*. Leaf evapotranspiration (E) was slightly modified by treatments at the beginning on the season (I × M, *p* ≤ 0.0001, Figure 3B) but no effect was observed later in the season. On the other hand, although no differences in instantaneous water use efficiency (WUE) were recorded at harvest, AMF inoculated plants showed a better WUE during berry development and ripening (Figure 3C). Finally, stomatal conductance (*g*_s_) was highly affected by the interaction between AMF inoculation and irrigation system during the whole season (Figure 3D). Thereby, AMF inoculation of HI plants mitigated the reduction of *g*_s_.

### Effect of AMF Inoculation and Irrigation Amounts on Merlot Berry Primary and Secondary Metabolites

Primary metabolites and berry fresh weight (BFW) are presented in Table 4. Must pH, TA, and TSS were not affected by treatments. However, BFW was modified by treatments; hence, AMF inoculation increased BFW of FI plants and decreased in HI (I × M, *p* = 0.05).

**TABLE 4:** Primary metabolites of Merlot/3309C grapevines subjected to different irrigation amounts (FI, Full Irrigated; HI, Half Irrigated), AMF inoculation (I, inoculated; NI, non-inoculated), and their combinations during the 2020 growing season (first productive year) in Oakville (California).

	Juice pH	TA (g/L)	TSS (°Brix)	BFW (g/berry)
**Treatments**				
FINI	3.31 ± 0.02	0.76 ± 0.03	25.43 ± 0.98	0.89 ± 0.06 ab
FII	3.32 ± 0.02	0.73 ± 0.01	25.08 ± 0.26	0.99 ± 0.05 a
HINI	3.31 ± 0.03	0.73 ± 0.02	26.48 ± 0.58	0.89 ± 0.02 ab
HII	3.32 ± 0.02	0.71 ± 0.05	24.60 ± 1.87	0.84 ± 0.03 b
***LMEM***				
Irrigation amount (I)	ns	ns	ns	.
AMF inoculation (M)	ns	ns	ns	.
I × M	ns	ns	ns	.

*Values represent means ± SE (n = 4) separated by Kenward–Roger method and Tukey’s p-value adjustment (P ≤ 0.05). Different letters within column, indicate significant differences as affected by Irrigation amount, I, AMF inoculation, M, and their interaction (I × M). ns indicate non-significance or significance at 10% probability level, respectively. BFW, berry fresh weight; LMEM, linear mixed-effect model.*

Flavonols and anthocyanins were monitored through berry ripening. The effect of AMF inoculation and irrigation systems on berry skin flavonol content and composition was modulated during the growing season as indicated by the significant interaction of treatments with the sampling dates (Supplementary Table 2). At mid ripening, the berry skin flavonol content increased in HINI grapevines (I × M, *p* ≤ 0.05, Table 5). Similarly, quercetin-3-*O*-glucoside and laricitrin-3-*O*-glucoside decreased with AMF inoculation under HI conditions (Table 5, I × M, *p* ≤ 0.05). At harvest, myricetin and quercetin derivatives were the most abundant flavonols found in Merlot berry skins, accounting for more than 40% of the total flavonols. Irrigation treatment was the main factor affecting flavonol content and composition as indicated by the decrease in quercetin, laricitrin, kaempferol, isorhanmetin, and syringetin derivative contents in HI grapevines (Table 5). It is noteworthy to highlight the increased content of quercetin-3-*O*-galactoside in HII grapevines (I × M, *p* ≤ 0.001).

**TABLE 5:** Berry skin flavonol content and composition of Merlot/3309C grapevines subjected to different irrigation amounts (FI, Full Irrigated; HI, Half Irrigated), AMF inoculation (I, inoculated; NI, non-inoculated), and their combinations during the 2020 growing season (first productive year) in Oakville (California).

*100% veraison*	Myricetin-3-*O*-galactoside	Myricetin-3-*O*-glucoside	Quercetin-3-*O*-galactoside	Quercetin-3-*O*-glucoside	Laricitrin-3-*O*-glucoside mg/g	Kaempferol-3-*O*-glucoside	Isorhamnetin-3-*O*-glucoside	Syringetin-3-*O*-glucoside	Total flavonols
**Treatments**									
FINI	0.027 ± 0.004	0.022 ± 0.001	0.235 ± 0.029	2.296 ± 0.145 ab	1.430 ± 0.235	0.152 ± 0.038	0.189 ± 0.064	0.020 ± 0.003	4.37 ± 0.28 ab
FII	0.025 ± 0.005	0.025 ± 0.008	0.245 ± 0.027	2.306 ± 0.125 ab	1.558 ± 0.026	0.174 ± 0.013	0.186 ± 0.037	0.024 ± 0.004	4.54 ± 0.21 ab
HINI	0.025 ± 0.005	0.016 ± 0.007	0.285 ± 0.024	2.564 ± 0.142 a	1.842 ± 0.046	0.214 ± 0.013	0.205 ± 0.047	0.028 ± 0.006	5.18 ± 0.34 a
HII	0.028 ± 0.008	0.025 ± 0.009	0.340 ± 0.115	2.052 ± 0.102 b	1.432 ± 0.050	0.178 ± 0.007	0.181 ± 0.033	0.023 ± 0.001	4.26 ± 0.29 b
***LMEM***									
Irrigation amount (I)	ns	ns	ns	ns	ns	⋅	ns	ns	ns
AMF inoculation (M)	ns	ns	ns	*	*	ns	ns	ns	*
I × M	ns	ns	ns	*	*	ns	ns	ns	*
***Harvest***									
**Treatments**									
FINI	0.18 ± 0.01	0.45 ± 0.04	0.20 ± 0.02 b	0.60 ± 0.07	1.49 ± 0.14	0.24 ± 0.02	0.16 ± 0.02	0.06 ± 0.01 ab	3.24 ± 0.46
FII	0.19 ± 0.02	0.53 ± 0.06	0.17 ± 0.02 b	0.63 ± 0.05	1.77 ± 0.18	0.27 ± 0.03	0.22 ± 0.03	0.07 ± 0.01 a	3.11 ± 0.27
HINI	0.17 ± 0.02	0.44 ± 0.05	0.12 ± 0.01 c	0.50 ± 0.06	1.28 ± 0.14	0.21 ± 0.02	0.14 ± 0.01	0.04 ± 0.00 bc	3.51 ± 0.47
HII	0.17 ± 0.02	0.41 ± 0.04	0.25 ± 0.03 a	0.55 ± 0.02	1.27 ± 0.15	0.19 ± 0.02	0.15 ± 0.02	0.03 ± 0.00 c	3.30 ± 0.08
***LMEM***									
Irrigation amount (I)	ns	ns	ns	*	*	*	*	***	*
AMF inoculation (M)	ns	ns	***	ns	ns	ns	ns	*	ns
I × M	ns	ns	***	ns	ns	ns	ns	*	ns

Berries were sampled at 100% veraison (July 28) and at harvest (August 26, 2020). Values represent means ± SE (n = 4) separated by Kenward–Roger method and Tukey’s p-value adjustment (P ≤ 0.05). Different letters within column, indicate significant differences as affected by Irrigation amount, I, AMF inoculation, M, and their interaction (I × M). ns, indicate non-significance. * and *** indicate non-significance or significance at 10, 5, and 0.1% probability levels, respectively. All values are expressed as mg of the compound per gram of skin dry weight. LMEM, linear mixed-effect model.

At mid ripening the main anthocyanin was cyanidin-3-*O*-glucoside, which accounted for *ca*. 20% (Table 6 and Supplementary Figure 4). The total anthocyanin content of Merlot berry skins was not affected by treatments but HI treatment decreased the contents of some anthocyanin derivatives (Table 6). At harvest, the total anthocyanin content in Merlot berry skins was not affected by different treatments (Table 6). Malvidin was the most abundant anthocyanin detected in Merlot berry skins (Supplementary Figure 5), with contents ranged between 23.1% for HINI plants and 28.7% from FII but none of the malvidin derivatives were affected by treatments (Table 6). The main changes in anthocyanin composition were due to irrigation treatments, thus, HI led to decreased contents of cyanidin and peonidin derivatives (I, *p* ≤ 0.05).

**TABLE 6:** Berry skin anthocyanin content and composition of Merlot/3309C grapevines subjected to different irrigation amounts (FI, full irrigated; HI, half irrigated), AMF inoculation (I, inoculated; NI, non-inoculated), and their combinations during the 2020 growing season (first productive year) in Oakville (California).

	3-Monoglucoside					3-Acetyl-glucoside					3-*p*-Coumaroyl-glucoside					Total
				anthocyanins												
			
*100% veraison*	Delphinidin	Cyanidin	Petunidin	Peonidin	Malvidin	Delphinidin	Cyanidin	Petunidin	Peonidin	Malvidin	Delphinidin	Cyanidin	Petunidin	Peonidin	Malvidin	
									mg/g							
**Treatments**																
FINI	1.06 a ± 0.12	1.22 ± 0.19	0.50 a ± 0.05	1.07 ± 0.12	0.71 ± 0.03	0.18 a ± 0.02	0.17 ± 0.03	0.11 ± 0.01	0.12 ± 0.01	0.14 ± 0.01	0.10 ± 0.01	0.16 a ± 0.01	0.04 ± 0.00	0.14 ± 0.01	0.06 ± 0.01	4.56 ± 0.19
FII	1.00 a ± 0.10	1.19 ± 0.14	0.48 a ± 0.04	1.02 ± 0.10	0.67 ± 0.05	0.17 a ± 0.01	0.14 ± 0.02	0.11 ± 0.01	0.12 ± 0.01	0.15 ± 0.00	0.10 ± 0.01	0.14 b ± 0.01	0.05 ± 0.00	0.13 ± 0.01	0.07 ± 0.00	5.36 ± 0.48
HINI	0.70 b ± 0.17	0.86 ± 0.27	0.34 b ± 0.07	0.78 ± 0.21	0.55 ± 0.09	0.12 ± 0.03	0.13 ± 0.04	0.08 ± 0.02	0.09 ± 0.02	0.11 ± 0.02	0.08 ± 0.02	0.13 b ± 0.03	0.04 ± 0.01	0.11 ± 0.03	0.05 ± 0.01	5.19 ± 1.18
HII	0.91 ab ± 0.08	1.06 ± 0.11	0.45 a ± 0.05	0.98 ± 0.07	0.69 ± 0.09	0.15 b ± 0.01	0.15 ± 0.01	0.10 ± 0.01	0.12 ± 0.01	0.14 ± 0.01	0.10 ± 0.02	0.16 a ± 0.01	0.05 ± 0.01	0.15 ± 0.01	0.06 ± 0.02	5.01 ± 0.62
***LMEM***																
Irrigation amount (I)	*	ns	.	ns	ns	*	ns	ns	ns	ns	ns	.	ns	ns	ns	ns
AMF inoculation (M)	ns	ns	ns	ns	ns	ns	ns	ns	ns	ns	ns	ns	ns	.	ns	ns
I × M	ns	ns	ns	ns	ns	ns	ns	ns	ns	ns	ns	.	ns	ns	ns	ns
***Harvest***																
**Treatments**																
FINI	7.54 ± 0.75	3.05 b ± 0.38	4.51 ± 0.36	3.85 ± 0.50	9.21 ± 0.52	1.20 ± 0.15	0.47 b ± 0.06	0.94 ± 0.10	0.43 b ± 0.04	2.32 ± 0.15	0.74 ± 0.08	0.36 b ± 0.04	0.53 ± 0.03	0.59 ± 0.06	1.63 ± 0.08	37.4 ± 4.5
FII	7.46 ± 0.76	3.79 a ± 0.48	4.40 ± 0.42	4.49 ± 0.52	9.06 ± 0.82	1.15 ± 0.10	0.52 a ± 0.05	0.89 ± 0.08	0.48 a ± 0.03	2.25 ± 0.22	0.74 ± 0.09	0.41 a ± 0.04	0.53 ± 0.05	0.66 ± 0.06	1.63 ± 0.19	38.5 ± 2.6
HINI	6.68 ± 0.79	2.43 c ± 0.13	4.08 ± 0.47	3.28 ± 0.43	8.87 ± 1.16	1.03 ± 0.13	0.34 c ± 0.03	0.83 ± 0.10	0.37 c ± 0.04	2.25 ± 0.31	0.67 ± 0.08	0.29 c ± 0.03	0.51 ± 0.06	0.52 ± 0.06	1.83 ± 0.07	33.8 ± 3.4
HII	6.87 ± 0.78	2.76 c ± 0.24	4.14 ± 0.47	3.67 ± 0.38	8.55 ± 1.12	1.10 ± 0.10	0.43 b ± 0.03	0.86 ± 0.09	0.38 c ± 0.03	2.12 ± 0.24	0.73 ± 0.09	0.33 b ± 0.03	0.49 ± 0.06	0.63 ± 0.05	1.40 ± 0.18	34.5 ± 2.8
***LMEM***																
Irrigation amount (I)	ns	*	ns	ns	ns	ns	*	ns	*	ns	ns	*	ns	ns	ns	ns
AMF inoculation (M)	ns	ns	ns	ns	ns	ns	ns	ns	ns	ns	ns	ns	ns	ns	ns	ns
I × M	ns	ns	ns	ns	ns	ns	ns	ns	ns	ns	ns	ns	ns	ns	ns	ns

Berries were sampled at 100% veraison (July 28) and at harvest (August 26, 2020). Values represent means ± SE (n = 4) separated by Kenward–Roger method and Tukey’s p-value adjustment (P ≤ 0.05). Different letters within column, indicate significant differences as affected by Irrigation amount, I, AMF inoculation, M, and their interaction (I × M). ns, indicate non-significance and * indicate non-significance or significance at 10%, and 5% probability levels, respectively. All values are expressed as mg of the compound per gram of skin dry weight. LMEM, linear mixed-effect model.

**FIGURE 4:**
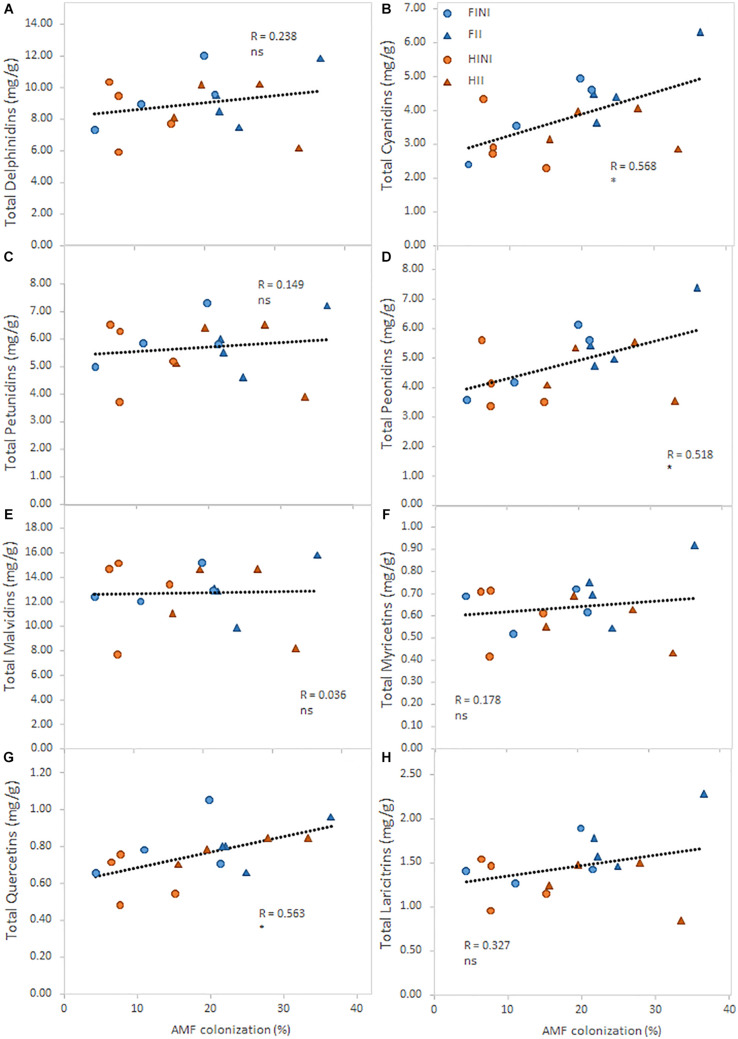
Relationships between total content of the main flavonoid group measured in berry skins and the incidence of the mycorrhizal colonization (%) of field grown Merlot/C3309 grapevines subjected to different irrigation amounts (FI, Full Irrigated; HI, Half Irrigated), AMF inoculation (I, inoculated; NI, non-inoculated), and their combinations. For each flavonoid compound, straight lines correspond to the linear regression lines fitted for the pooled data of all treatments, ns and * indicate non-significance and significance at 5% probability levels, respectively.

Finally, an analysis of the relationship between the percentage of AMF colonization and the main flavonoid contents was conducted (Figure 4). The intensity of the AMF colonization had a significant positive relationship with total cyanidins (Figure 4B; *R* = 0.57; *p* ≤ 0.05), total peonidins (Figure 4D; *R* = 0.52; *p* ≤ 0.05), and total quercetins (Figure 4G; *R* = 0.56; *p* ≤ 0.05).

### Analysis of the Economic and Environmental Profitability of Cultural Practices

Analysis of the cost of implementing the different treatments in vineyards indicated that HI irrigation led to decreased yields per hectare (Table 7, I, *p* ≤ 0.05). However, the reduction in yield did not lead to a significant diminution of the gross income per hectare (I, *p* = 0.873). Regarding their impact of water resources, HI irrigation system increased the green component of the WF and decreased the blue WF (I, *p* ≤ 0.05, and *p* ≤ 0.001, respectively). Therefore, HI contributed to a decrease of total WF in the first productive year of Merlot vineyard.

**TABLE 7:** Cost estimates on labor operations (Kurtural et al., 2020) and water footprint (Zotou and Tsihrintzis, 2017) of Merlot/3309C grapevines subjected to different irrigation amount (FI, full irrigated; HI, half irrigated), AMF inoculation (I, inoculated; NI, non-inoculated), and their combinations during the 2020 growing season (first productive year) in Oakville (California).

Labor operation cost	AMF inoculation ($/Ha)	Irrigation ($/Ha)	Total ($/Ha)	Yield (kg/Ha)	Gross income ($/Ha)
**Treatment**					
FINI	0	529.25	529.25	528.3 a ± 50.0	264.5 ± 30.6
FII	15	529.25	544.25	428.3 b ± 50.0	104.5 ± 47.1
HINI	0	363.86	363.86	410.0 b ± 16.7	251.1 ± 30.2
HII	15	363.86	378.86	348.3 c ± 33.3	127.4 ± 22.5
***LMEM***					
Irrigation amount (I)	–	–	–	*	ns
AMF inoculation (M)	–	–	–	ns	ns
I × M	–	–	–	ns	ns
Water footprint (WF)	Green WF (m^3^/ton)	Blue WF (m^3^/ton)	Gray WF (m^3^/ton)	Total WF (m^3^/ton)	
**Treatment**					
FINI	1061.7 c ± 121.5	1706.1 b ± 195.3	ND	2767.8 b ± 316.8	
FII	1331.3 ab ± 180.9	2139.2 a ± 290.7	ND	3470.5 a ± 471.5	
HINI	1326.7 b ± 65.2	1065.8 d ± 52.4	ND	2392.5 c ± 117.6	
HII	1597.7 a ± 152.5	1283.6 c ± 122.5	ND	2881.3 b ± 275.0	
***LMEM***					
Irrigation amount (I)	*	***	–	⋅	
AMF inoculation (M)	ns	ns	–	ns	
I × M	ns	ns	–	ns	

Values represent means ± SE (n = 4) separated by Kenward–Roger method and Tukey’s p-value adjustment (P ≤ 0.05). Different letters within column, indicate significant differences as affected by Irrigation amount, I, AMF inoculation, M, and their interaction (I × M). ns, indicate non-significance, * and *** indicate non-significance or significance at 10, 5, and 0.1% probability levels, respectively. All values are expressed as mg of the compound per gram of skin dry weight. LMEM, linear mixed-effect model; ND, not determined.

## Discussion

In the last decades, warming trends in viticulture areas have been described worldwide (Petrie and Sadras, 2008; Fraga et al., 2013; Hannah et al., 2013; Neethling et al., 2017). Likewise, weather data recorded during 2020 growing season in Oakville, CA, United States (Figure 1), suggested more stressful conditions for grapevines comparing to the average of last 20 years, challenging their production and quality. Indeed, a recent study based on climate indices suggested a reduction of 8,700 km^2^ for the California land suitable for grapevine cultivation by mid-21st century (Monteverde and De Sales, 2020). Within this scenario, smart-farming techniques are mandatory for adaptation and mitigation to guarantee the future of the winemaking industry and for reducing potential water conservation issues.

### AMF Inoculation and Irrigation Amounts Modulated Water Status, Photosynthetic Performance, Growth, and Mineral Content of Young Merlot Vines

Colonization analysis of Merlot grapevine roots indicated that AMF inoculated integrated with the native communities colonizing grapevine roots (Table 1). Thus, we found that the percentage of mycorrhizal colonization was two to three fold higher in mycorrhizal inoculated treatments compared to non-inoculated ones. However, no differences in mycorrhizal colonization due to water amount received by plants were evident in accordance with a previous study conducted on fruit-bearing cuttings (Torres et al., 2018d). In contrast, a study conducted on own-rooted Cabernet Sauvignon field grapevines reported increased frequency of arbuscules and reduced fine root production when an additional water deficit was applied to the regulated deficit irrigation (RDI) plot, suggesting that plants could compensate the lower density of fine roots in vines facing water deficit by increasing AMF colonization (Schreiner et al., 2007). These discrepancies between studies may be explained by the fact that grapevines responded to the degree of water deficit from the previous growing season. Thus, Schreiner et al. (2007) observed increased arbuscular colonization at bloom, before the onset of differences between the treatments they applied whereas under our experimental conditions, water amounts received by Merlot grapevines the previous season did not differ. AMF colonization data also confirmed the seasonality effect on mycorrhizal colonization (Nogales et al., 2009) and the reinforcement that AMF inoculation exerts on native mycorrhizal colonization (Nicolás et al., 2015). Without imposed water stress, AMF inoculation impaired vegetative growth as indicated in the RMD index. However, when grapevines were subjected to HI treatment, AMF-inoculated vines grew better as indicated by the RMD, green pruning, and trunk diameter. Nevertheless, leaf area was not enhanced after AMF inoculation according to previous studies (Nogales et al., 2019), which would explain that AMF inoculation was not sufficient to avoid the yield loss due to HI treatment.

It is well established that AMF inoculation enhances mineral nutrition of grapevines presumably by a greater exploration of soil by the external hyphal network of the AMF resulting in more efficient roots for obtaining nutrients from soils (Smith and Read, 2008). Moreover, it was recently reported that the inoculation of grapevines with AMF under controlled conditions led to the upregulation of nutrient transport genes (Balestrini et al., 2017). In spite of the consensus about AMF enhancing grapevine nutrient uptake, contradictory results are reported about increased mineral nutrient content due to the symbiosis (Nicolás et al., 2015; Torres et al., 2018a). Leaf or petiole mineral nutrient content might be useful for the diagnostic of soil mineral deficiencies allowing growers to manage them. However, concentration of mineral nutrients does not provide accurate information on nutrient uptake or allocation of nutrient in various organs (Schreiner, 2016). Therefore, although no differences on the mineral nutrient content in leaf blades were observed, mineral uptake was presumably enhanced by AMF inoculation given the growth promotion recorded in mycorrhizal plants under HI conditions. Furthermore, Balestrini et al. (2017) recently reported that although mineral nutrient uptake genes were upregulated after inoculation with different inoculants (*F. mosseae* vs. a fungal and bacterial consortium), the degree of upregulation differed between them, suggesting a specific response to a specific inoculum. Similarly, Nogales et al. (2019) did not find accumulation of minerals in grapevine leaves after AMF inoculation with the exception of P, which was enhanced and decreased after *F. mosseae* and *R. irregulare* inoculations, respectively.

Grapevine water status monitored during the growing season showed that irrigation amounts were the main factor affecting the plant water status. Thus, according to previous work FI plants were maintained under well-watered conditions with values of midday SWP higher than −0.9 MPa (van Leeuwen et al., 2009) and/or *g*_s_ higher than 200 mmol m^–2^ s^–1^ (Medrano et al., 2002). On the other hand, grapevines subjected to HI were not exposed to a severe water stress as they never reached values of SWP and *g*_s_ lower than −1.5 MPa and 50 mmol m^–2^ s^–1^, respectively, considered detrimental for grapevine development (Medrano et al., 2002; Villalobos-González et al., 2019).

We did not measure any SWP differences due to the AMF inoculation when plants were subjected to FI. However, within HI plants, AMF inoculation tended to result in higher SWP values (Figure 2) in accordance with previous studies (Nicolás et al., 2015). Therefore, a higher AMF occurrence in the root zone has been related to improve water status of vines by increasing water uptake presumably by increasing the mycorrhizal structures, mainly arbuscules (Schreiner et al., 2007). Accordingly, we observed that photosynthetic performance of AMF inoculated Merlot grapevines was improved (namely, *A*_N_ or WUE) (Figure 3). Likewise, Nicolás et al. (2015) found a better photosynthetic performance after inoculating Crimson grapevines grown in a commercial vineyard. Indeed, a recent meta-analysis demonstrated that AMF exert a positive influence on photosynthetic rates, stomatal conductance, and water use efficiency on both C3 and C4 plants subjected to salt stress (Chandrasekaran et al., 2019).

### Flavonoid Composition of Berry Skins From Young Merlot Grapevines Is Modulated by AMF and Irrigation Amounts

Merlot grapevines did not show changes on their berry primary metabolites as affected by the treatments applied (Table 4). Similarly, a recent study evaluating the effect of different sustained deficit irrigation (SDI) and RDI showed no differences in must pH and TSS in Merlot berries in a 4-year field experiment conducted in a hot climate (Munitz et al., 2017). This lack of effect of the irrigation systems on berry primary metabolism might be due to grapevines were not subjected to a severe water stress (discussed above). On the other hand, previous studies showed that inoculation with AMF of grapevines vineyards did not affect TSS or TA under field conditions (Nicolás et al., 2015) or under controlled conditions (Torres et al., 2018d, 2019) and our results corroborated these findings.

Regarding secondary metabolism, neither irrigation systems nor AMF inoculation modified flavonol and anthocyanin total content at harvest (Tables 5, 6). Similarly, a 2-year field study conducted in Central valley in California with Merlot did not report differences on flavonol or anthocyanin skin content due to different irrigation amounts (Yu et al., 2016). A previous study conducted on Cabernet Sauvignon subjected to water deficit reported that although flavonol synthesis related genes were up-regulated after the onset of fruit ripening, this did not affect berry flavonol concentration at harvest (Castellarin et al., 2007). Similarly, previous studies with Tempranillo grown under controlled conditions did not observed differences due to AMF inoculation on the total content of flavonol and anthocyanins in berry skins (Torres et al., 2019).

Flavonol composition was affected by treatments. Thus, HII grapevines increased quercetin and decreased syringetin contents in berry skins at harvest in accordance to a previous study (Torres et al., 2019). Indeed, it is known that AMF inoculation up-regulated phenyl-propanoid biosynthesis key genes in grapevines in response to pathogens (Bruisson et al., 2016). On the other hand, HI led to decreased contents of quercetins, laricitrins, kaempferols, syringetins, and isorhamnetins. Likewise, Martínez-Lüscher et al. (2014) found that in spite of the increase in *O*-methyl-transferase (OMT) transcript level, methylated flavonols (i.e., isorhamnetins, laricitrins, and syringetins) did not increase under water deficit. These authors suggested that given the higher affinity of OMT for quercetins, the lower concentration of quercetins under water deficit could act as a limiting factor for the synthesis of methylated forms, and our findings corroborated this hypothesis.

Regarding anthocyanin composition, berry skins from HI grapevines showed lower contents of di-substituted anthocyanins (cyanidin and peonidin derivatives) than the ones of FI grapevines. It is well known that water deficit regulates the expression of key genes of the flavonoid pathway such as the flavonoid 3′-hydroxylase, flavonoid 3′,5′-hydroxylase, and *O*-methyltransferase in red cultivars (Castellarin et al., 2007; Deluc et al., 2009). Therefore, these decreased contents of di-substituted anthocyanins were likely explained by a different regulation of these genes when grapevines are subjected to water deficit.

The role of AMF for enhancing phenolic compounds was reported in several studies with potted grapevines. Thus, AMF grapevines showed increased content of resveratrol, viniferins, and pterostilbene (Bruisson et al., 2016), total phenols and quercetin content (Eftekhari et al., 2012), and total flavonoids (Torres et al., 2018a) in leaves of different grapevine varieties facing different biotic and/or abiotic stresses. Moreover, increased anthocyanin contents were reported in berries from grapevines grown under water deficit and warming conditions (Torres et al., 2018d). Similarly, we found a strong relationship between the percentage of mycorrhizal colonization and some flavonoids (Figure 4).

The economic analysis data indicated that AMF inoculation and water management did not affect the cost of labor operations, in spite of irrigating with half amount may lead to decreases in yield. However, this came with reductions of the water footprint that have to be taken into account. It is noteworthy that extreme weather recorded in 2020 could modulate the effects described in this work. Moreover, the mycorrhizal extraradical mycelium coexists with soil microbial communities and the synergistic activity between the AMF, the bacterial communities, and the grapevine modulates the benefits of symbiosis on nitrogen fixation, P solubilization, and production of phytohormones, siderophores, and antibiotics (Giovannini et al., 2020). On the other hand, previous studies demonstrated that the microbiome of vineyards is shaped by cropping management (Coller et al., 2019), and little is known about whether these communities stimulate or suppress the extraradicular mycelium activity (Svenningsen et al., 2018). Therefore, given the effect of AMF inoculation and different irrigation amounts had on grapevine physiology and berry composition, further studies should consider the potential effects of these management practices on vineyard soil living microbiota.

## Conclusion

Current research aimed to study how Merlot grapevines responded to AMF inoculation and different water amounts in their first productive year *in situ*. Our results highlighted the role of AMF inoculation for improving vegetative growth, photosynthetic activity, and water status of grapevines, especially when facing mild water deficits in field grown grapevines. Additionally, a strong relationship between the mycorrhizal colonization of roots and some flavonoids was found, corroboration the effect of AMF for regulating anthocyanin and flavonol metabolisms. Finally, although some berry quality traits and grapevine performance (i.e., water status or gas exchange parameters) were improved by AMF inoculation under water deficit, AMF inoculation was not sufficient to avoid the yield losses due to water deficit in the first productive year of Merlot when facing a hyper-arid growing season. It is noteworthy that these results may be affected by edaphoclimatic characteristics and living microbiota in vineyard soils, which should be taken into account before making the decision of inoculating the vineyard. Therefore, this study offer a starting point to assess the effect of AMF inoculation on young vines under real field conditions.

## Data Availability Statement

The raw data supporting the conclusions of this article will be made available by the authors, without undue reservation.

## Author Contributions

NT and SK conceived the project. SK acquired the funding. NT and SK designed the project. NT curated the data and wrote the first version of the manuscript. RY curated and proofed the data. All authors approved the final version of the manuscript.

## Conflict of Interest

The authors declare that the research was conducted in the absence of any commercial or financial relationships that could be construed as a potential conflict of interest.

## Footnotes

1 www.valent.com/Products/1f42d59a-c1fd-4d1c-b6a2-8c4a6486cc81/mycoapply-endomaxx
