# Supplementary material for: Arbuscular Mycrorrhizal Fungi Inoculation and Applied Water Amounts Modulate the Response of Young Grapevines to Mild Water Stress in a Hyper-Arid Season
Source: Front Plant Sci. 2021 Jan 14;11:622209. doi: 10.3389/fpls.2020.622209 (PMC7840569; doi:10.3389/fpls.2020.622209)

Table S1. F value and associated probability calculated with the linear mixed-effect model with AMF inoculation (M), irrigation (I), sampling point (T) and their combinations as fixed factors, and replicate as random factor for AMF colonization, stem water potential (SWP), net carbon assimilation (A_N_), leaf evapotranspiration (E), instantaneous water use efficiency (WUE) and stomatal conductance (g_s_). All parameters were measured in Oakville (California) during the 2020 growing season on field grown Merlot/3309C grapevines inoculated or not and subjected to different irrigation treatments in their first productive year.

|  |  | **Mycorrhizal inoculation (M)** | | **Irrigation (I)** | | **Sampling point (T)** | | **M × I** | | **M × T** | | **I × T** | | **M × I × T** | |
| --- | --- | --- | --- | --- | --- | --- | --- | --- | --- | --- | --- | --- | --- | --- | --- |
| **Linear mixed-effect model** | | F value | Pr(>F) | F value | Pr(>F) | F value | Pr(>F) | F value | Pr(>F) | F value | Pr(>F) | F value | Pr(>F) | F value | Pr(>F) |
| Mycorrhizal colonization (%) | | 0.11 | 0.737 | 0.36 | 0.552 | 92.80 | **≤0.0001** | 0.06 | 0.807 | 17.68 | **≤0.0001** | 1.62 | 0.208 | 0.01 | 0.916 |
| SWP (-MPa) | | 0.24 | 0.624 | 0.23 | 0.632 | 12.54 | **0.001** | 0.41 | 0.521 | 0.01 | 0.972 | 3.98 | 0.048 | 0.09 | 0.755 |
| A_N_ (µmol m^-2^ s^-1^) | | 2.98 | 0.085 | 0.01 | 0.956 | 22.52 | **≤0.0001** | 4.12 | **0.043** | 1.86 | 0.173 | 1.72 | 0.19 | 3.09 | 0.079 |
| E (mmol m^-2^ s^-1^) | | 0.03 | 0.867 | 0.91 | 0.341 | 6.28 | **0.012** | 0.01 | 0.91 | 0.06 | 0.806 | 0.44 | 0.503 | 0.05 | 0.815 |
| WUE(A/E) | | 11.84 | **0.001** | 1.43 | 0.232 | 7.13 | **0.008** | 14.48 | **≤0.0001** | 6.88 | 0.0095 | 0.74 | 0.388 | 8.52 | **0.004** |
| g_s_ (mmol m^-2^ s^-1^) | | 0.34 | 0.559 | 0.17 | 0.683 | 6.75 | **0.009** | 0.21 | 0.642 | 1.68 | 0.195 | 6.78 | **0.009** | 2.20 | 0.138 |

Table S2. . F value and associated probability calculated with the linear mixed-effect model with AMF inoculation (M), irrigation (I), sampling point (T) and their combinations as fixed factors, and replicate as random factor for flavonol compounds and total flavonols (mg/g berry dried skin). All parameters were measured in Oakville (California) during the 2020 growing season on field grown Merlot/3309C grapevines inoculated or not and subjected to different irrigation treatments in their first productive year.

|  | **Mycorrhizal inoculation (M)** | | **Irrigation (I)** | | **Sampling point (T)** | | **M × I** | | **M × T** | | **I × T** | | **M × I × T** | |
| --- | --- | --- | --- | --- | --- | --- | --- | --- | --- | --- | --- | --- | --- | --- |
| **Linear mixed-effect model** | F value | Pr(>F) | F value | Pr(>F) | F value | Pr(>F) | F value | Pr(>F) | F value | Pr(>F) | F value | Pr(>F) | F value | Pr(>F) |
| Myricetin-3-O-galactoside | 0.40 | 0.533 | 3.12 | **0.086** | 22.83 | **≤0.0001** | 0.57 | 0.455 | 0.58 | 0.450 | 1.41 | 0.242 | 0.65 | 0.425 |
| Myricetin-3-O-glucoside | 2.44 | 0.128 | 5.36 | **0.027** | 27.77 | **≤0.0001** | 3.71 | 0.062 | 1.48 | 0.231 | 2.47 | 0.125 | 2.06 | 0.161 |
| Quercetin-3-O-galactoside | 4.68 | 0.038 | 0.00 | 0.956 | 4.87 | **0.034** | 3.46 | **0.071** | 0.94 | 0.337 | 1.56 | 0.219 | 0.85 | 0.363 |
| Quercetin-3-O-glucoside | 0.05 | 0.814 | 1.22 | 0.275 | 96.49 | **≤0.0001** | 0.01 | 0.920 | 8.18 | 0.007 | 0.7 | 0.406 | 4.93 | **0.034** |
| Laricitrin-3-O-glucoside | 0.61 | 0.440 | 9.05 | **0.005** | 10.52 | **0.002** | 1.42 | 0.241 | 1.47 | 0.233 | 8.82 | **0.006** | 0.54 | 0.465 |
| Kaempferol-3-O-glucoside | 1.66 | 0.206 | 7.15 | **0.011** | 3.44 | **0.072** | 1.95 | 0.172 | 0.05 | 0.824 | 9.8 | **0.004** | 0.01 | 0.907 |
| Isorhamnetin-3-O-glucoside | 0.58 | 0.452 | 4.38 | **0.045** | 3.81 | **0.061** | 1.52 | 0.226 | 0.01 | 0.914 | 2.66 | 0.114 | 0.31 | 0.581 |
| Syringetin-3-O-glucoside | 7.51 | **0.009** | 42.39 | **≤0.0001** | 5.81 | **0.021** | 9.55 | **0.004** | 1.58 | 0.216 | 27.27 | **≤0.0001** | 2.21 | 0.147 |
| Total flavonols | 0.15 | 0.696 | 7.82 | **0.008** | 25.23 | **≤0.0001** | 0.65 | 0.423 | 2.53 | 0.121 | 7.55 | **0.009** | 1.07 | 0.308 |

Table S3. F value and associated probability calculated with the linear mixed-effect model with AMF inoculation (M), irrigation (I), sampling point (T) and their combinations as fixed factors, and replicate as random factor for anthocyanin compounds and total anthocyanins (mg/g berry dried skin). All parameters were measured in Oakville (California) during the 2020 growing season on field grown Merlot/3309C grapevines inoculated or not and subjected to different irrigation treatments in their first productive year.

|  | **Mycorrhizal inoculation (M)** | | **Irrigation (I)** | | **Sampling point (T)** | | **M × I** | | **M × T** | | **I × T** | | **M × I × T** | |
| --- | --- | --- | --- | --- | --- | --- | --- | --- | --- | --- | --- | --- | --- | --- |
| **Linear mixed-effect model** | F value | Pr(>F) | F value | Pr(>F) | F value | Pr(>F) | F value | Pr(>F) | F value | Pr(>F) | F value | Pr(>F) | F value | Pr(>F) |
| 3-Monoglucoside | |  |  |  |  |  |  |  |  |  |  |  |  |  |
| Delphinidin | 0.10 | 0.751 | 0.22 | 0.636 | 26.25 | **≤0.0001** | 0.08 | 0.779 | 0.00 | 0.993 | 0.54 | 0.468 | 0.00 | 0.999 |
| Cyanidin | 0.26 | 0.610 | 0.79 | 0.381 | 2.98 | **0.090** | 0.20 | 0.661 | 0.20 | 0.659 | 2.34 | 0.137 | 0.74 | 0.396 |
| Petunidin | 0.07 | 0.784 | 0.12 | 0.727 | 33.46 | **≤0.0001** | 0.06 | 0.81 | 0.01 | 0.991 | 0.45 | 0.509 | 0.00 | 0.965 |
| Peonidin | 0.23 | 0.634 | 0.31 | 0.582 | 9.77 | **0.004** | 0.17 | 0.679 | 0.05 | 0.822 | 1.67 | 0.205 | 0.35 | 0.559 |
| Malvidin | 0.03 | 0.862 | 0.02 | 0.899 | 36.00 | **≤0.0001** | 0.02 | 0.878 | 0.09 | 0.756 | 0.18 | 0.674 | 0.05 | 0.832 |
| 3-Acetyl-glucoside | |  |  |  |  |  |  |  |  |  |  |  |  |  |
| Delphinidin | 0.07 | 0.790 | 0.06 | 0.807 | 23.56 | **≤0.0001** | 0.06 | 0.815 | 0.11 | 0.745 | 0.67 | 0.419 | 0.12 | 0.734 |
| Cyanidin | 0.34 | 0.566 | 0.20 | 0.654 | 3.04 | **0.090** | 0.44 | 0.511 | 0.08 | 0.785 | 3.24 | 0.081 | 0.03 | 0.867 |
| Petunidin | 0.05 | 0.821 | 0.05 | 0.815 | 30.16 | **≤0.0001** | 0.03 | 0.86 | 0.06 | 0.797 | 0.49 | 0.488 | 0.13 | 0.726 |
| Peonidin | 0.74 | 0.393 | 0.25 | 0.618 | 14.83 | **≤0.0001** | 0.57 | 0.454 | 0.86 | 0.358 | 4.31 | 0.045 | 1.35 | 0.254 |
| Malvidin | 0.01 | 0.924 | 0.02 | 0.892 | 37.23 | **≤0.0001** | 0.00 | 0.951 | 0.11 | 0.732 | 0.13 | 0.713 | 0.03 | 0.861 |
| 3-p-Coumaroyl-glucoside | | |  |  |  |  |  |  |  |  |  |  |  |  |
| Delphinidin | 0.03 | 0.856 | 0.04 | 0.849 | 25.51 | **≤0.0001** | 0.03 | 0.868 | 0.18 | 0.676 | 0.22 | 0.645 | 0.11 | 0.747 |
| Cyanidin | 0.89 | 0.351 | 0.08 | 0.784 | 3.57 | **0.060** | 0.83 | 0.368 | 0.16 | 0.692 | 3.15 | 0.085 | 0.57 | 0.457 |
| Petunidin | 0.04 | 0.846 | 0.01 | 0.919 | 32.55 | **≤0.0001** | 0.01 | 0.905 | 0.14 | 0.709 | 0.35 | 0.553 | 0.07 | 0.791 |
| Peonidin | 0.37 | 0.545 | 0.03 | 0.873 | 15.48 | **≤0.0001** | 0.26 | 0.607 | 0.00 | 0.966 | 1.44 | 0.239 | 0.13 | 0.721 |
| Malvidin | 0.00 | 0.962 | 0.01 | 0.941 | 29.81 | **≤0.0001** | 0.00 | 0.989 | 0.38 | 0.537 | 0.09 | 0.764 | 0.23 | 0.634 |
| Total anthocyanins | 0.11 | 0.740 | 0.16 | 0.692 | 26.86 | **≤0.0001** | 0.09 | 0.772 | 0.04 | 0.838 | 0.77 | 0.386 | 0.07 | 0.790 |

**Figure S1**. Mycorrhizal structures from Merlot/3309C roots observed with the Stereoscope Microscope (A) and with the microscope optique 40X (B).


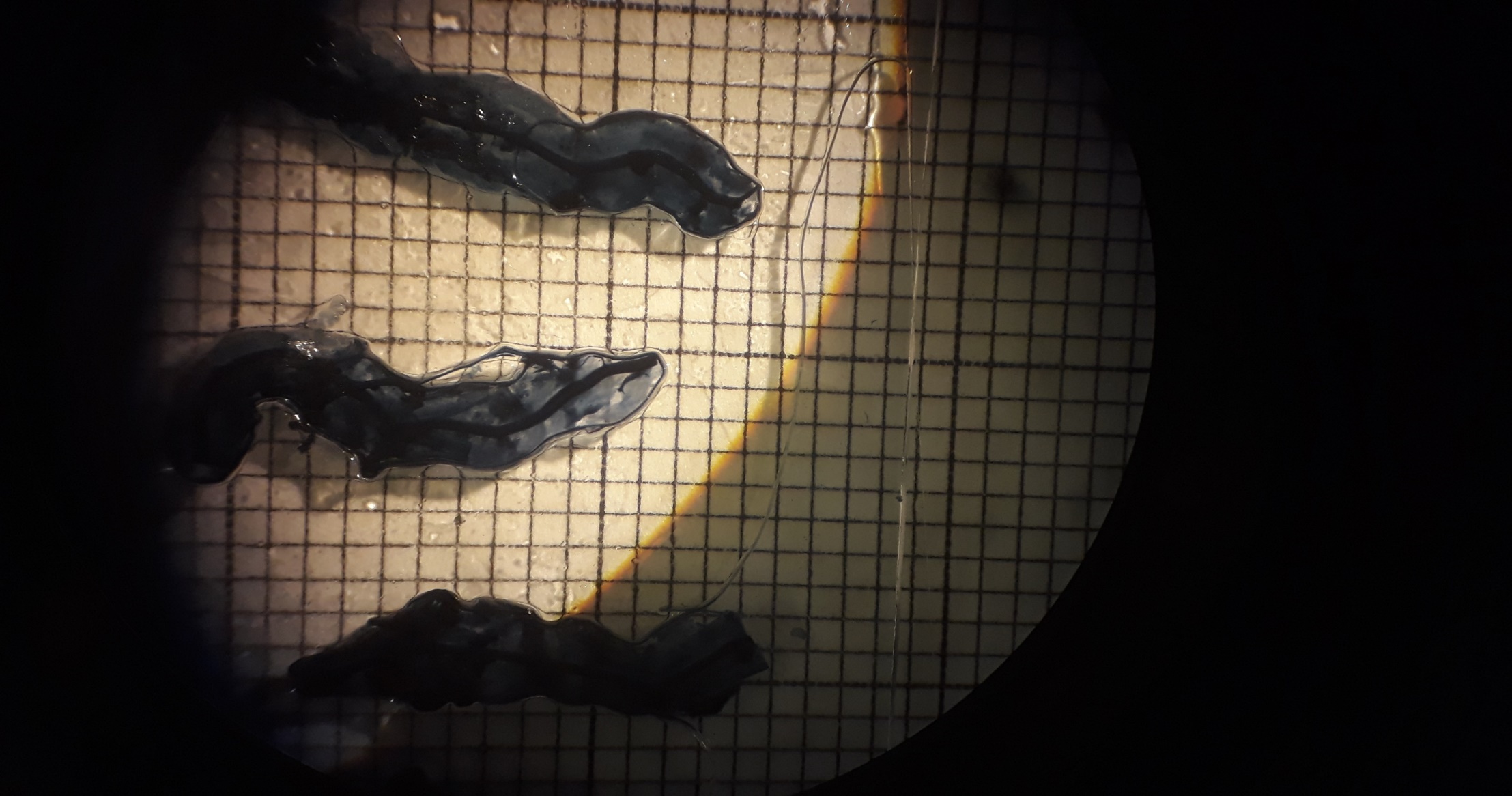

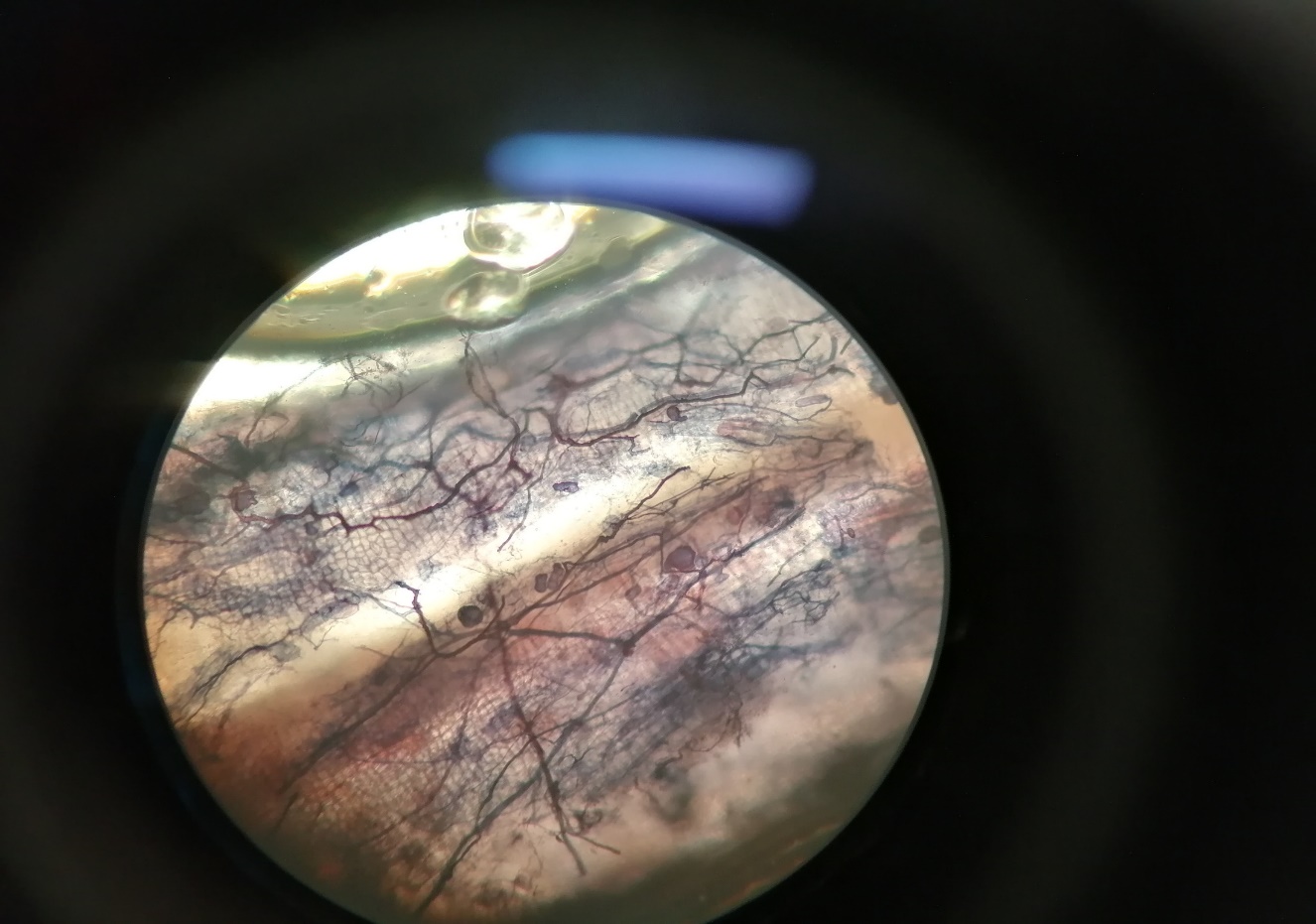


**A**

**B**

**Figure S2**. HPLC chromatogram showing the Merlot/3309C berry skin flavonol profile at veraison detected at 365 nm. Myr-3-glucU: Myricetin-3-O-glucuronide; Myr-3-glc: Myricetin-3-O-glucoside; Que-3-gal: Quercetin-3-O-galactoside; Que-3-glucU: Quercetin-3-O-glucuronide; Que-3-glc: Quercetin-3-O-glucoside; Lar-3-glc: Laricitrin-3-O-glucoside; Kaemp-3-glc: Kaempferol-3-O-glucoside; Isor-3-glc: Isorhamnetin-3-O-glucoside; Syr-3-glc: Syrigenin-3-O-glucoside.
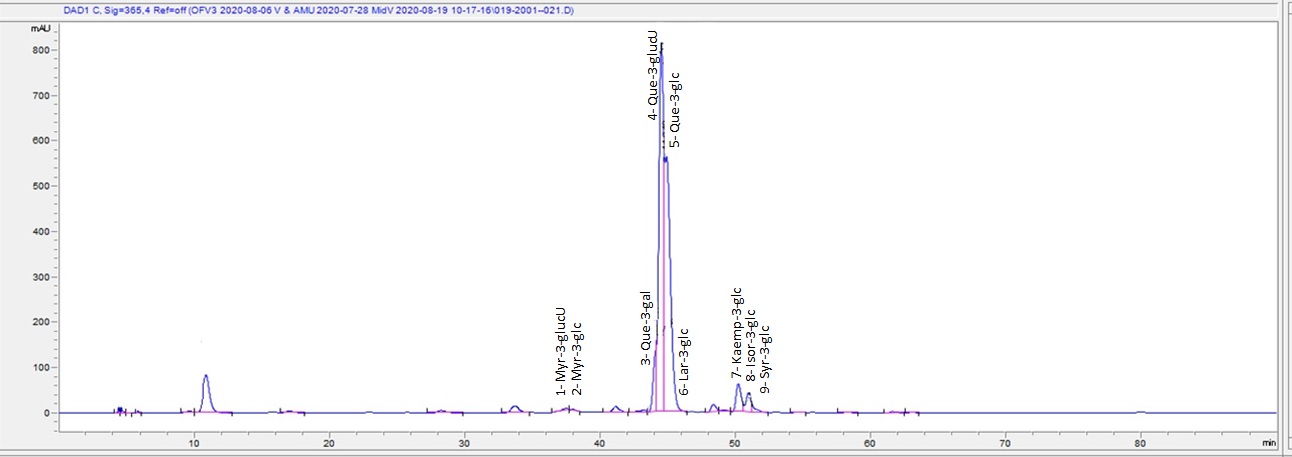


**Figure S3**. HPLC chromatogram showing the Merlot/3309C berry skin flavonol profile at harvest detected at 365 nm. Myr-3-glucU: Myricetin-3-O-glucuronide; Myr-3-glc: Myricetin-3-O-glucoside; Que-3-gal: Quercetin-3-O-galactoside; Que-3-glucU: Quercetin-3-O-glucuronide; Que-3-glc: Quercetin-3-O-glucoside; Lar-3-glc: Laricitrin-3-O-glucoside; Kaemp-3-glc: Kaempferol-3-O-glucoside; Isor-3-glc: Isorhamnetin-3-O-glucoside; Syr-3-glc: Syrigenin-3-O-glucoside.


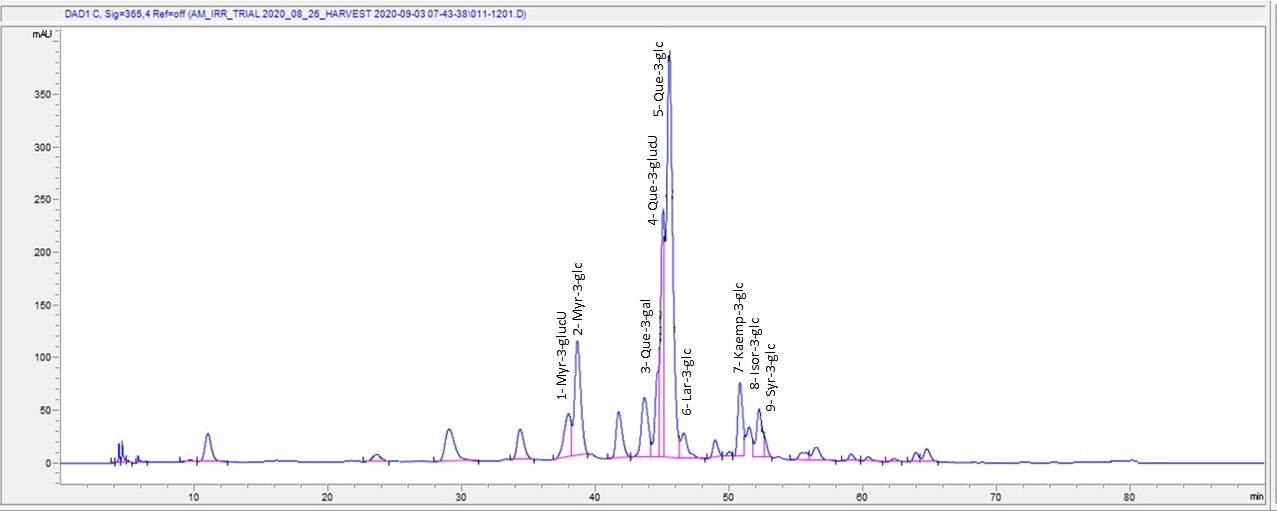


**Figure S4**. HPLC chromatogram showing the Merlot/3309C berry skin anthocyanin profile at veraison detected at 520 nm. Dp-3-glc: Delphinidin-3-glucoside; Cy-3-glc: Cyanidin-3-glucoside; Pt-3-glc: Petunidin3-glucoside; Pn-3-glc: Peonidin-3-glucoside; Mv-3-glc: Malvidin-3-glucoside; Dp-3-glc-ac: Delphinidin-3-acetyl-glucoside; Cy-3-glc-ac: Cyanidin-3-acetyl-glucosides; Pt-3-glc-ac: Petunidin-3-acetyl-glucoside; Pn-3-glc-ac: Peonidin-3-acetyl-glucoside; Dp-3-glc-cou: Delphinidin-3 p-coumaroyl-glucoside; Mv-3-glc-ac: Malvidin-3-acetyl-glucoside; Cy-3-glc-cou: Cyanidin-3 p-coumaroyl-glucoside; Pn-3-glc-cou: Peonidin-3 p-coumaroyl-glucoside; Pt-3-glc-cou: Petunidin-3 p-coumaroyl-glucoside; Mv-3-glc-cou: Malvidin-3 p-coumaroyl-glucoside.


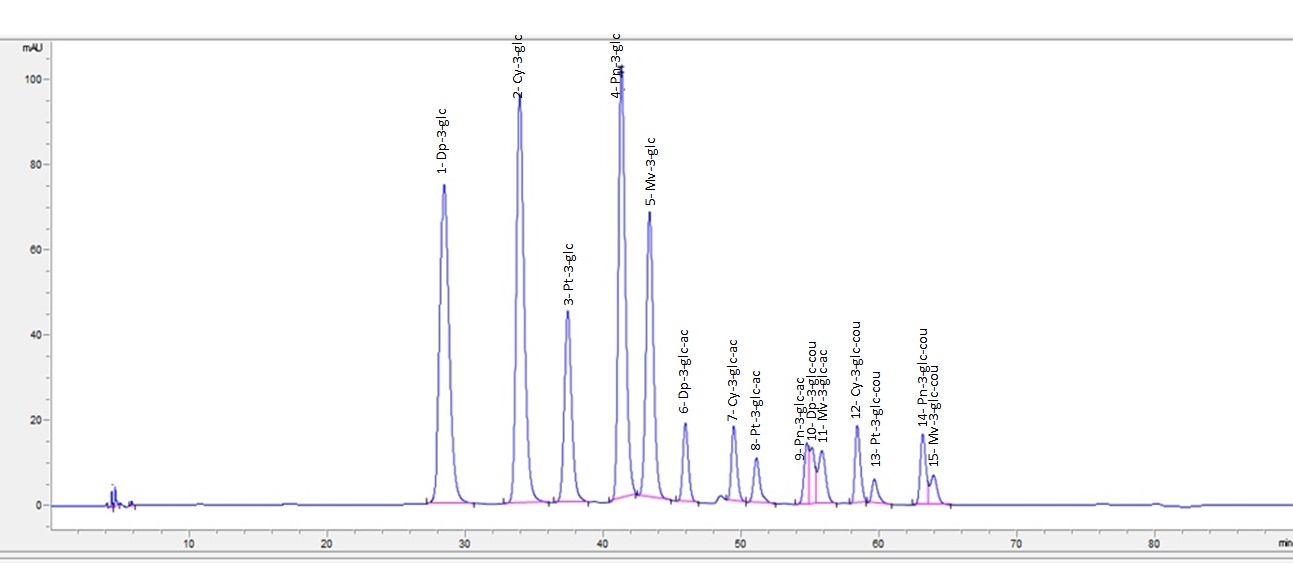


**Figure S5**. HPLC chromatogram showing the Merlot/3309C berry skin anthocyanin profile at harvest detected at 520 nm. Dp-3-glc: Delphinidin-3-glucoside; Cy-3-glc: Cyanidin-3-glucoside; Pt-3-glc: Petunidin3-glucoside; Pn-3-glc: Peonidin-3-glucoside; Mv-3-glc: Malvidin-3-glucoside; Dp-3-glc-ac: Delphinidin-3-acetyl-glucoside; Cy-3-glc-ac: Cyanidin-3-acetyl-glucosides; Pt-3-glc-ac: Petunidin-3-acetyl-glucoside; Pn-3-glc-ac: Peonidin-3-acetyl-glucoside; Dp-3-glc-cou: Delphinidin-3 p-coumaroyl-glucoside; Mv-3-glc-ac: Malvidin-3-acetyl-glucoside; Cy-3-glc-cou: Cyanidin-3 p-coumaroyl-glucoside; Pn-3-glc-cou: Peonidin-3 p-coumaroyl-glucoside; Pt-3-glc-cou: Petunidin-3 p-coumaroyl-glucoside; Mv-3-glc-cou: Malvidin-3 p-coumaroyl-glucoside.


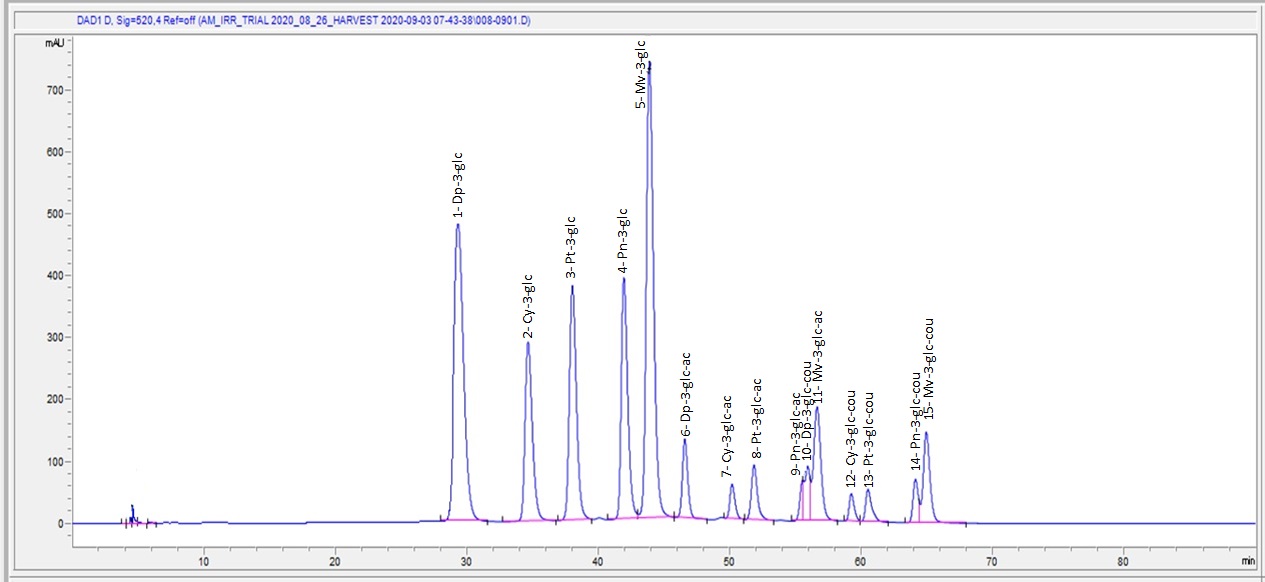

Supplement: Supplementary file 1 [file Data_Sheet_1.docx]
